# Social Determinants of Health in People Living with Psychiatric Disorders: The Role of Pharmacists

**DOI:** 10.1089/heq.2022.0189

**Published:** 2023-04-19

**Authors:** Carla D. Cobb, Shari N. Allen, Joseph M. Cusimano, Michelle Ding, Amanda S. Eloma, Carol A. Ott, Kimberly B. Tallian

**Affiliations:** ^1^ Capital Consulting, Billings, Montana, USA.; ^2^ PCOM Georgia School of Pharmacy, Suwanee, Georgia, USA.; ^3^ Pharmacy Practice Department, Bernard J. Dunn School of Pharmacy, Shenandoah University, Winchester, Virginia, USA.; ^4^ Kaiser Permanente, Los Angeles, California, USA.; ^5^ Kings County Hospital, NYC Health + Hospitals, Brooklyn, New York, USA.; ^6^ Department of Pharmacy Practice, Purdue University/Eskenazi Health, Indianapolis, Indiana, USA.; ^7^ Scripps Mercy Hospital, San Diego, CA, San Diego, California, USA.

**Keywords:** clinical pharmacists, community pharmacists, psychiatric disorders, public health, social determinants of health, healthy people programs

## Abstract

**Introduction:**

Social determinants of health (SDOH) affect outcomes of people living with psychiatric disorders, including substance use disorders. As experts in medication optimization, pharmacists play a vital role in identifying and addressing medication-related problems associated with SDOH. However, there is a paucity of literature on how pharmacists can be part of the solution.

**Objective:**

The purpose of this article is to provide a narrative review and commentary on the intersection between SDOH, medication-related outcomes in people living with psychiatric disorders, and the role of pharmacists in addressing them.

**Method:**

The American Association of Psychiatric Pharmacists appointed an expert panel to research the issue, identify barriers, and develop a framework for including pharmacists in addressing medication therapy problems associated with SDOH in people with psychiatric disorders. The panel used Healthy People 2030 as the framework and sought input from public health officials to propose solutions for their commentary.

**Results:**

We identified potential connections between SDOH and their impact on medication use in people with psychiatric disorders. We provide examples of how comprehensive medication management can afford opportunities for pharmacists to mitigate medication-related problems associated with SDOH.

**Conclusion:**

Public health officials should be aware of the vital role that pharmacists play in addressing medication therapy problems associated with SDOH to improve health outcomes and to incorporate them in health promotion programs.

## Background

The World Health Organization (WHO) defines social determinants of health (SDOH) as “the nonmedical factors that influence health outcomes.” They are the conditions in which people are born, grow, work, live, and age as well as the wider set of forces and systems shaping the conditions of daily life.^1^ This article will explore the interaction between SDOH, psychiatric disorders, and the role of pharmacists in the United States. The definitions and frameworks of SDOH have been described elsewhere by a number of organizations, including WHO and Healthy People 2030.^1,2^ Healthy People 2030 will serve as the framework for the discussion in this article.

SDOH have significant effects on patient-level health outcomes, including morbidity, mortality, and quality of life.^3,4^ A study showed that nonmedical factors or social determinants account for 80–90% of a person's health.^5^ SDOH can also impact medication-related outcomes.^6^ Pharmacists have long been aware of challenges that patients often face in accessing prescription medications, including medication cost, lack of or limited insurance coverage, or distance from a pharmacy.^7^ Pharmacists are trained in providing direct patient care for people living with chronic conditions, including psychiatric disorders, by identifying and resolving medication therapy problems (MTPs).^8^ With more pharmacists practicing in team-based medical practices, additional SDOH that impact or intersect with optimal medication use, beyond access to medications, have emerged.^9^ Applying a SDOH framework can serve as a foundation for understanding this intersection and the role that pharmacists can play in addressing medication problems related to SDOH faced by patients.

Psychiatric disorders are common and undertreated. Twenty-one percent of adults in the United States live with a mental illness (52.9 million people in 2020), with functional impairment ranging from mild to severe.^10^ According to the 2020 National Drug Use and Health Survey done by the Substance Abuse and Mental Health Service Administration (SAMHSA), 46.2% of those received mental health (MH) services in the past year. In 2020, 6.7% of adults (9.5 million) had both a mental illness and substance use disorder (SUD) but only 5.7% of these received treatment for both conditions.^11^

MTPs are common in people with psychiatric disorders, such as complex medication regimens, inadequate treatment of co-occurring medical conditions, and inadequate monitoring for metabolic adverse effects.^12^ Low medication adherence rates, common in psychiatric disorders, may also be related to SDOH.^13^ The identification of MTPs through comprehensive medication management (CMM), an individualized approach to identifying and solving MTPs, provides a number of opportunities for pharmacists to recognize and mitigate SDOH-related barriers.^14^ CMM is the process of care used by pharmacists who provide direct patient care. CMM assesses all of a patient's medications for indication, effectiveness, safety (adverse effects, drug interactions), and adherence. The purpose of CMM is to identify and resolve MTPs to optimize the patient's medication regimen toward their treatment goals.

## Methods

There is a paucity of literature on the connection between SDOH, psychiatric disorders, and the role of pharmacists. With this in mind, The American Association of Psychiatric Pharmacists commissioned an expert panel to explore this intersection. This article should not be considered a systematic review but rather a narrative review and commentary on the topic with recommendations for future work and research. The expert panel met monthly for over 1 year to describe, research, and develop a framework for the topic and to propose barriers and opportunities for involving pharmacists in addressing this issue. We also engaged two public health professionals to provide feedback on article drafts for relevance to a public health audience.

**FIG. 1.**
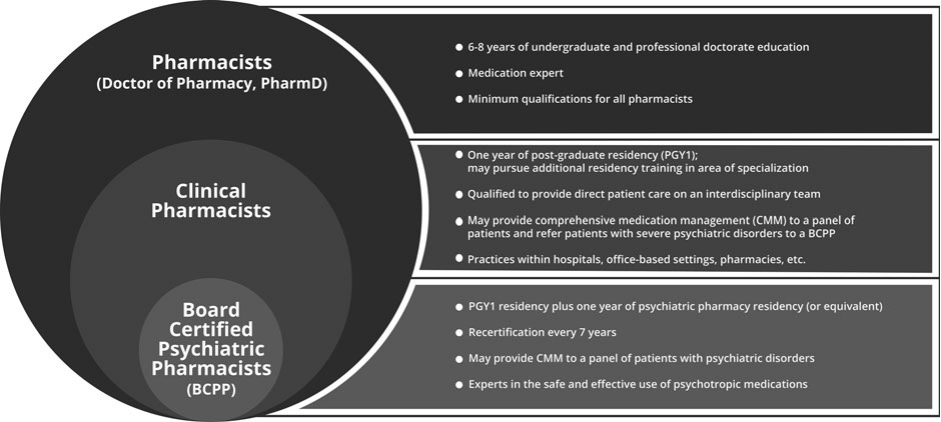
Overview of pharmacist training and specialization in the United States. The diagram provides a simplified overview of the credentials, training, and practice settings for pharmacists, with a focus on distinguishing clinical pharmacists and psychiatric pharmacists from other pharmacists. This diagram does not represent all forms of pharmacy practice, as pharmacists practice in several other settings, including hospital pharmacy administration, industry, and academia. The Doctor of Pharmacy (PharmD) has been the entry-level educational credential for pharmacists since 2000, requiring a total of 6–8 years of undergraduate and professional education. Community pharmacists, practicing in the outpatient, retail setting in either chain or independent pharmacies, are essential providers of medication advice and are responsible for the safe and effective dispensing of medications pursuant to legally valid prescriptions. Clinical pharmacists, whose roles are frequently divorced from traditional dispensing responsibilities, are generally board certified in their specialty areas, have completed 1–2 years of postgraduate residency training, and may practice in either the inpatient (hospital) or outpatient (ambulatory care) settings. Some pharmacists are board certified in pharmacotherapy or ambulatory care pharmacy while others have expertise in psychiatry or 1 of the other 11 BPS-recognized specialties. Community pharmacists, while not required, may also be board certified and residency trained. BPS, Board of Pharmacy Specialties.

The focus of this article is on the role of clinical pharmacists, defined as “pharmacists who engage in the direct observation and evaluation of the patient and their medication-related needs; the initiation, modification, or discontinuation of patient-specific pharmacotherapy; and the ongoing pharmacotherapeutic monitoring and follow-up of patients in collaboration with other health professionals.”^15^ The role of community pharmacists and psychiatric pharmacists are also discussed. Please see Figure 1 for a description of pharmacist training and specialization.

## SDOH and Psychiatric Disorders

Healthy People 2030 grouped the SDOH into five domains: economic stability, education access and quality, health care access and quality, neighborhood and built environment, and social and community context.^2^ The organization identified areas within the five domains that may have an impact on psychiatric disorders in addition to other medical conditions. Within the domain of economic stability, unemployment may cause negative psychiatric consequences such as reported feelings of depression, anxiety, low self-esteem, demoralization, and worry.^2^ Overcrowded housing and being unsheltered may also affect MH, stress levels, interpersonal relationships, and sleep.^16,17^

Special consideration should also be given when prescribing medications for unsheltered people with psychiatric disorders to ensure optimal outcomes.^18^ In terms of education access and quality, poor education is associated with poorer physical and MH such as smoking, unhealthy diet, and substance misuse. In contrast, higher educational attainment is associated with better social outcomes, lower morbidity, and later mortality.^17^

However, an individual's current state of MH and educational attainment can be bidirectional where possessing mental wellbeing is predictive of college success.^16^ Children from low-income families, with disabilities, and who routinely experience social discrimination are more likely to have psychiatric disorders, such as depression.^16,17^ Also, the stress of living in poverty can affect children's brain development.^16,17^ Children exposed to violence, neglect, or abuse may exhibit behavior problems, depression, anxiety, post-traumatic stress disorder, and increased signs of aggression.^16,17^ These effects may be carried into adulthood and can result in greater risk for substance use.^16,17^

Individuals who have limited health care access due to lack of or inadequate insurance coverage or transportation may not receive timely and quality psychiatric services.^16,17^ This limited access may leave their conditions inadequately managed by delaying care, skipping medications, and missing appointments.^16,17^ Within the social and community domain, individuals with a history of incarceration are in worse mental and physical health compared with the general population.^16,17^

Decreasing social isolation and belonging to groups can improve MH.^17^ There is also evidence of social discrimination related to reduced access to pharmacies in Black and Latino neighborhoods,^19^ lower rates of prescribing medications for treating opioid use disorders in Black and Hispanic areas,^20^ and higher rates of prescribing potent first-generation antipsychotics, with serious adverse effects, to Black patients.^21^

Table 1 lists SDOH that affect MH as categorized by Compton and Shim, who describe the significance of these SDOH in increasing risk of poor MH, increased morbidity, and early mortality.^17^ These social determinants are closely related to the five domains of SDOH described in Healthy People 2030.^2^

**Table 1.** Social determinants of mental health

Racial discrimination and social exclusion	Adverse early life experiences
Poor education	Unemployment, underemployment, and job insecurity
Poverty, income inequality	Neighborhood deprivation
Poor access to sufficient healthy food	Poor housing quality and instability
Adverse features of the built environment	Poor access to health care and transportation
Exposure to violence, conflict, and war	Mass incarceration and poor relations between law enforcement and communities
Poor environmental quality	Discrimination (nonracial)
Adverse features of the workplace	

Adapted from Compton and Shim.^17^

Fisher and Baum discuss the potential biological mechanisms behind how certain social determinants may negatively impact physical or MH by acting as stressors and triggering the arousal of neural and somatic stress responses in the body.^16^ Chronic stress arousal may increase the risk of psychiatric disorders such as depressive and anxiety disorders or SUD.^16^

There is a clear link between SDOH and psychiatric disorders but there is a lack of literature on the role that pharmacists can play in identifying and mitigating the impact of SDOH on medication use in people with psychiatric disorders. This article aims to describe barriers and opportunities to engage pharmacists in potential solutions to this issue.

### Current barriers

Although there is increasing awareness of the intersection between SDOH, MH, and the role of pharmacists, there are still significant barriers in addressing these factors.^9^

We identified two articles that studied the role of pharmacists and SDOH. A systematic literature review revealed that medication adherence was impacted by two SDOH: food insecurity and unstable housing.^22^ Pestka et al. evaluated the experience of pharmacists incorporating SDOH into CMM visits where they used a process for uncovering SDOH, relying on open-ended questions and structured documentation.^9^ Little other research, however, has addressed this important issue.

Epic, an electronic medical record system used by multiple health care systems across the United States, has incorporated a SDOH tool allowing users to collect SDOH data. However, this tool is underutilized.^23^ A barrier may be lack of perceived time by health care providers as they are collecting other data for billing, diagnosis, and/or preventative care. While these data may be collected by nonhealth care counterparts (i.e., foodbanks, shelters), lack of interoperability between electronic medical record systems results in underutilization of SDOH for health care outcomes.^23^

Historically, SDOH had not been widely integrated into pharmacy school curricula. The Accreditation Council for Pharmacy Education updated their standards in 2016 to include SDOH, and while many schools of pharmacy now incorporate diversity, equity, and inclusion or cultural competency initiatives into their curriculum, not all domains of SDOH are routinely incorporated into the curriculum.^24,25^ The absence of SDOH in the curriculum then limits SDOH consideration in the postgraduate pharmacy residency programs.

Chandra surveyed pharmacy residents and program directors in residency programs at U.S. Department of Veterans Affairs medical centers to assess their knowledge of SDOH.^25^ In their routine patient care, no residents and a limited number of residency program directors asked patients about SDOH, such as food insecurity. Of the survey participants, few were familiar with or had received training in Healthy People 2020 initiatives in their residency program.^25^ In conjunction with SDOH, pharmacists benefit from training in adverse childhood experiences (ACEs), trauma informed care, and shared decision making. It is well documented that ACEs contribute to negative health outcomes.^26^

In addition to the limited SDOH in training, there may also be minimal awareness by health care professionals about appropriate follow-up. If patients are asked about the domains of SDOH as a standard patient care practice, there should also be follow-up. The health care professional should be able to address a patient's needs not only by incorporating the collected data into their patient care but also by referring them to appropriate services. Failure to do so may widen this gap.

These gaps in awareness, research, education, and practice of addressing SDOH may result in poor outcomes for those with psychiatric disorders. Recognition and utilization of SDOH in the health care system will help to stop this cycle.

## The Role of Pharmacists Today

Given the importance of SDOH on health outcomes, it is imperative for pharmacists who provide care to people with psychiatric disorders to consider the intersection of SDOH and medication use (Table 2).^27^ There are opportunities for pharmacist involvement within each of the five domains, although the role of the pharmacist may differ by practice area and specialty. The following sections elaborate on the role of pharmacists today in recognizing and addressing MTPs related to SDOH in people with psychiatric disorders.

**Table 2.** Social determinants of health and medication use

SDOH domain	Potential impact on medication use
Economic stability	• Medical: medication affordability, prescription insurance plan selection, and benefit utilization • Access to technology • Nutrition: food–drug interactions, dietary management of medical conditions, food insecurity and medication adherence, access to healthy food (e.g., food swamps/food deserts), medication/food administration requirements, cultural and religious influences (e.g., religious fasting)
Neighborhood and built environment	• Pharmacy deserts, access to medications (transportation barriers) • Healthy housing (e.g., air quality and ventilation, mold, rodents, or pests), housing instability, medication adherence • Public safety • Seniors' independent living • Telehealth infrastructure • Facilities for physical activity (e.g., parks, gyms)
Education access and quality	• Medical literacy/education, including health behaviors/change • Technology literacy (e.g., internet, telehealth services)
Social and community context	• Support groups and medication education groups • Culture around medication taking • Foster care • Faith-based organizations and other charitable organizations
Health care access and quality	• Access to care, including long-acting injectable medications (for patients with low adherence),^44^ clozapine (treatment-resistant schizophrenia),^46^ pharmacogenomic testing, access to the opioid antagonist naloxone, medication-assisted treatment for opioid use disorder • Pharmacist prescriptive authority • Safe psychotropic use in pregnancy • Measurement-based care • Beliefs about health care, vaccinations, and medications

*Source:* Healthy People 2030: SDOH Literature Summaries.^27^

SDOH, social determinants of health.

### Community pharmacists

Community (or retail) pharmacists play a key role in identifying and mitigating SDOH for patients with psychiatric disorders. While most Americans live within 5 miles of a community pharmacy, there are still many Americans living in “pharmacy deserts” (defined as urban areas in which at least half of the population lives more than 1 mile from the nearest pharmacy) and in rural counties without pharmacies or in which the nearest pharmacy is more than 10 miles away, indicating that there is still a need to improve access to pharmacy services to all Americans.^19,28,29^ People with serious mental illness (SMI) may be particularly disadvantaged by a long trip to the pharmacy, with one study of Kansas Medicaid beneficiaries with SMI noting substantial transportation barriers, including being unable to drive safely due to adverse effects from medications.^30^ If trained, community pharmacists can identify SDOH related to medication use, and develop and implement (in collaboration with partners from other disciplines) individualized care plans.

Community pharmacists' services extend beyond their important medication-dispensing role, such as providing evidence-based consultation on the use of psychotropics in pregnancy,^31^ screening for depression,^32^ administering long-acting injectable antipsychotics,^33^ and assisting older adults with navigating Medicare.^34^ By virtue of the public's trust and general proximity to pharmacists in the community, pharmacists can help navigate barriers to optimal medication use related to SDOH.^35^

### Clinical pharmacists

Clinical pharmacists have a broad role in identifying and mitigating SDOH in their care of people with psychiatric disorders. CMM, an individualized approach to identifying and resolving MTPs,^36^ requires clinical pharmacists to incorporate SDOH into recommendations. An analysis of patient medication-related needs (indication, effectiveness, safety, and adherence) by SDOH domain is presented in Table 3. “Indication” refers to the appropriate use of medication to treat a specific condition. “Effectiveness” refers to how well a medication works for a specific disease state. “Safety” addresses the risks of medications, such as adverse effects and drug interactions. “Adherence” refers to whether a patient can take a medication as indicated. Clinical pharmacists can solve MTPs across every SDOH domain.

**Table 3.** Examples of social determinants of health×indication, effectiveness, safety, and adherence crosswalk

	Indication	Effectiveness	Safety	Adherence
Economic stability	Reduce prescription costs by deprescribing unnecessary (and potentially harmful) therapies	Reduce missed workdays due to untreated psychiatric disorder (e.g., optimizing dosing of antidepressants)	Prevent unnecessary hospitalizations due to adverse events (e.g., mitigating the drug–drug interaction between valproic acid products and lamotrigine to prevent Stevens–Johnson syndrome)	Addressing prescription cost concerns (e.g., resolving insurance issues, connecting people with patient assistance programs) can facilitate adherence Addressing barriers to adherence can lower overall health care costs
Education access and quality	Educate on the utility of medications to treat common psychiatric disorders like alcohol use disorder	Optimizing ADHD treatment can prevent loss of educational attainment	Teach adolescents about the harms of cannabis use	Consult with parents regarding daily use of stimulants and discuss risk versus benefits of “medication holidays”
Health care access and quality	Screen for psychiatric disorders like ADHD, depression, and anxiety disorders and refer to a primary care physician Advocate for and use statewide protocols to expand access to effective smoking cessation treatment	Collaborative practice agreements (e.g., manage pharmacotherapy for major depressive disorder for patients enrolled in a mood disorder clinic)	Ensure timely monitoring for metabolic disturbances secondary to antipsychotic use	Connect patients with outpatient pharmacies that will provide adherence packaging (i.e., “bubble packs”) Provide long-acting injectable medication administration
Neighborhood and built environment	Provide means for safe needle disposal with pamphlets to connect people who inject drugs to treatment	Use motivational interviewing to assist with smoking cessation	Train people who inject drugs to administer naloxone to reduce fatal drug overdoses in the community	Encourage adherence to prevent the social sequelae of untreated mental illness (e.g., damage to relationships) Medication delivery services
Social and community context	Refer patients in need of social support (e.g., providing contact information for local grief counseling after the death of a loved one)	Manage opioid use disorder with buprenorphine, reducing the impact of substance abuse on the community	Distribute timer caps and lock boxes to prevent accidental overdose	Provide CMM, improving health literacy Patient medication education groups Tailor medication handouts to the reading level and culture of the audience

ADHD, attention-deficit/hyperactivity disorder; CMM, comprehensive medication management.

### Psychiatric pharmacists

Psychiatric pharmacists have additional education, training, and/or experience that provides unique expertise in the treatment of people with psychiatric disorders, including the medication-related challenges they face.

There are over 1500 psychiatric pharmacists positioned to help address the psychiatric provider shortage by increasing the capacity of the system to care for more patients with psychiatric disorders.^37,38^ It has been predicted that the psychiatrist shortfall will be about 27% by 2030.^39^ Psychiatric pharmacists' ability to function under a collaborative practice agreement and provide CMM has been endorsed by the National Council for Mental Wellbeing Medical Director Institute.^39^ As members of a primary care team, psychiatric pharmacists can extend access to evidence-based expertise about psychotropic medications^41^ and improve provider comfort with psychotropic prescribing.^42^

However, psychiatric pharmacists continue to be underutilized.^38^ Psychiatric pharmacists can improve access to care while being mindful of SDOH issues, including the application of pharmacogenetics, measurement-based care, advanced psychopharmacology, population health, and evidence-based medicine for treatment of psychiatric disorders. They can help identify and incorporate the patient's goals of care into the treatment plan, coordinate care for psychiatric and medical disorders during active treatment and care transitions, as well as educate other providers, peers, trainees, patients, families, and caregivers. The lack of federal recognition as providers under the Social Security Act is a major barrier to patient access to psychiatric pharmacist services.^43^

It has been estimated that more than two-thirds of individuals with schizophrenia are nonadherent with their antipsychotics, and within 6 months of being discharged from the hospital 50% discontinue their antipsychotics.^44,45^ Reasons include cost, adverse effects, and difficulty navigating the health care system, including prescription refills.^44^ Nonadherence impacts SDOH, as people with untreated schizophrenia may struggle to maintain employment or meet daily needs like securing food and maintaining hygiene.^46^ Strategies to improve medication adherence for people with schizophrenia include improving access to long-acting injectable antipsychotics, providing patient and caregiver education about the importance of taking medications, monitoring for side effects, recommending personalized adherence strategies, and addressing cost concerns.

Psychiatric pharmacists can support the use of clozapine, an underutilized antipsychotic, effective for treatment-resistant schizophrenia but associated with serious side effects and drug interactions.^47^ Psychiatric pharmacists can identify appropriate patients, and provide vital education and monitoring.^48,49^ By improving adherence to psychiatric medications, psychiatric pharmacists can play a critical role as a member of the health care team.

Psychiatric pharmacists also address key SDOH barriers for people with SUDs, including access to quality care. Access to opioid use disorder programs is a key factor in the opioid crisis,^50^ and psychiatric pharmacists help by writing medication-assisted treatment protocols (e.g., to facilitate buprenorphine induction in emergency departments) and facilitating continuation of medication-assisted treatment during transitions of care.^51^ Psychiatric pharmacists also participate in harm reduction, educating the public, furnishing naloxone kits, and naloxone administration training.^52^ Addressing substance use can help improve the wellness of families and communities, beyond each individual impacted patient.

## The Role of the Pharmacists Tomorrow

At the patient level, pharmacists are well positioned to conduct screenings for SDOH, identify unmet needs, and make referrals.^53^ The advent of community resources platforms (e.g., *NowPow* [nowpow.com], *Unite Us* [uniteus.com]) has simplified the search and referral process to local food pantries, health insurance carriers, and other community services. Other recommendations for pharmacists' engagement in addressing SDOH are outlined below.

### Education

Until recently SDOH has not been a focus in pharmacy education. Coursework has traditionally taught the therapeutic use of medications, but attention to SDOH is an area that deserves greater focus. The CMM categories of appropriateness, effectiveness, adherence, and safety can be viewed through a SDOH lens when reviewing drug therapy regimens (Table 2). Examples include medication cost, adverse effects, dosing, and treatment necessity (economic stability/food insecurity); understanding medication information and adherence (education access and quality), and medication effectiveness and monitoring (health care access and quality). Interprofessional education emphasizes and recognizes the role of various disciplines in team-based patient-centered care and is an opportunity to incorporate the principles of CMM with SDOH.

Pharmacists should also be trained in the evaluation of ACEs, their effect on health, and models of prevention and treatment with an emphasis in trauma-informed care.^54^ ACEs are important to understand as they have been shown to impact how an individual interacts with and trusts the health care system. A high number of ACEs can lead to disadvantages in economic stability and education access that potentially decrease health literacy, medication adherence, and ability to afford medications and health care. When issues related to SDOH are identified, pharmacists should collaborate with team members to address disparities and provide referrals for resources.

Psychiatric pharmacy residency training includes training on effective interactions with patients, family members, and caregivers related to empathy, cultural competence, and patient empowerment for their responsibility for their health. Psychiatric pharmacy residency training should also include SDOH application experiences that provide an understanding of the impact of SDOH on the ability of the individual patient to address and manage their MH. SDOH content has been added to the national certification exam for psychiatric pharmacists, ensuring that they are competent in this domain.

The concept of shared decision making is at the root of patient-centered care. The ability to apply shared decision making can help the clinical pharmacist assess the patient's personal goals, desire for treatment, and identification of SDOH barriers. Pharmacists should be trained in and encouraged to practice shared decision making as a standard of clinical practice.^55^

### Advocacy

Public health professionals can collaborate with pharmacists to advocate at the community level. Pharmacists provide a valuable perspective on medication and health care outcomes that may influence policy decisions. Many community boards and advocacy groups have open meetings^56^ and allocate time for public comment. Pharmacists may serve as board members, join grassroots movements, and/or local advocacy groups. Pharmacists may also be integral partners with local and state health departments for community health needs assessments. Establishing community partnerships, forming alliances with local agencies, and engaging key stakeholders can harness the knowledge and understanding necessary to develop programs and strengthen services that align with community culture. Pharmacist-led community outreach can be tailored for subpopulations to maximize impact (e.g., medication safety, diabetes education, chronic disease management).

At the state and federal level, pharmacists should be recognized and empowered to contribute toward health policy development and legislation. Additional contributions may be made through administrative positions in overseeing health programs and workgroups designed to improve public health through programmatic interventions (e.g., National Diabetes Prevention Program [cdc.gov/diabetes/prevention], Health Literacy Workgroup [health.gov], Racial and Ethnic Approaches to Community Health [REACH, cdc.gov/nccdphp/dnpao/state-local-programs/reach]). Legislation strengthening practice at a level consistent with pharmacists' education and training and supporting reimbursement for pharmacists' services should be reinforced. Pharmacists may also consider conducting SDOH research with PhenX Toolkit (phenxtoolkit.org), a publicly available database funded by the National Institutes of Health.

Advocacy related to MH issues is a current priority at local, state, and federal levels. Psychiatric pharmacists bring patient-level expertise related to MH and treatment that can inform policy interventions. Pharmacists who are not familiar with advocacy and policy roles can access toolkits such as the University of Kansas Community Toolbox (ctb.ku.edu) to influence outcomes and participate in policy discussions.

At the national level, pharmacists can advocate for congressional action primarily through membership and activity in pharmacy professional organizations such as the American Association of Psychiatric Pharmacists (formerly known as the College of Psychiatric and Neurologic Pharmacists, cpnp.org). As the voice of psychiatric pharmacists, AAPP has taken a public position calling for the elimination of federal X-waivers for buprenorphine prescribing to increase access to medication-assisted treatment,^57^ shown support for the Mom's Matter Act to “expand access to treatments and support for maternal psychiatric disorders for individuals from racial and ethnic minority groups,”^57^ and advocated for expanding access to psychiatric care in rural areas by ensuring equitable reimbursement for telehealth by Medicare.^55^

Pharmacists who work with patients living with psychiatric disorders should be encouraged to reflect upon their practices and translate their experiences with the health disparities that people with psychiatric disorders often face into a passion for advocacy.

Pharmacists have the ability to impact SDOH on every level such as incorporation into patient care practice, serving on health system committees, and influencing health policy. As stated during 1986 Ottawa Charter for Health Promotion: “Health promotion goes beyond health care. It puts health on the agenda of policymakers in all sectors and at all levels, directing them to be aware of the health consequences of their decisions and to accept their responsibilities for health.”^58^

### Future research

Research into the role of pharmacists in SDOH should expand beyond medication adherence, effectiveness, and safety to include the availability and usefulness of screening tools for SDOH in electronic health records in the clinical pharmacy practice setting, how these tools are used, and what role pharmacists should play in addressing SDOH to improve health outcomes. Research into how pharmacists in the direct patient care setting use SDOH screenings to determine barriers during patient appointments can inform how subsets of the population are impacted by health disparities that may be addressed by the pharmacist or should be referred to another member of the health care team.

There is currently limited published research related to pharmacists and SDOH focused on psychiatric disorders. Research related to pharmacy education should expand on the effectiveness of teaching methods, including interprofessional education and active learning strategies, to improve student pharmacist understanding and comfort with assessing individual patient SDOH and evaluating available resources, helping to address SDOH barriers. Future research should include public health experts in the design and evaluation process. A limitation of this article is that it was authored solely by psychiatric pharmacists, which may have limited the perspective of the article, but feedback from two public health professionals was included.

## Conclusion

Pharmacists play a critical role in recognizing and mitigating the impact of SDOH on medication use in patients with psychiatric disorders. Pharmacists are trained to provide direct, patient-centered care, allowing them to practice in a variety of health care settings, including in community pharmacies, clinic settings, and hospitals. Pharmacists are frequently at the front lines of SDOH-related obstacles to optimal medication use, such as cost and lack of insurance. However, SDOH related to home and food insecurity as well as transportation issues may not be readily identified. Healthy People 2030 describes broad SDOH, including employment status, poverty, safe housing, history of incarceration, and social discrimination that significantly impact people's ability to maximize the effectiveness of medical interventions, including optimal use of medications.

Pharmacists should not only be able to identify SDOH, but also incorporate SDOH into follow-up plans of care. Collaboration with other clinicians, including social workers, is an important aspect of follow-up care to ensure that referrals to resources are addressed by the care team. As such, pharmacists would benefit from additional training and education to identify and mitigate these SDOH obstacles.

In addition to SDOH training, pharmacists, in partnership with public health professionals, can advocate for policies and programs at the local, regional, and national level that address health equity and recognize health disparities that impact diverse patient populations. This includes attention not only to challenges in health and health systems but also to the areas where people live, learn, work, and play. Public health professionals should engage pharmacists as integral team members in developing and implementing health promotion programs that seek to improve the lives of communities.

Lastly, more research is needed to identify how pharmacists can directly address SDOH in people with psychiatric disorders and what the overall impact of these interventions has on medication-related outcomes.

SDOH affect medication-related outcomes in people living with psychiatric disorders. Pharmacists have a critical role in identifying and addressing modifiable barriers with SDOH and MTPs. Many communities suffer from a shortage of psychiatric providers. Psychiatric pharmacists are uniquely positioned to mitigate the impact of medication-related SDOH through their expertise in providing CMM for patients with psychiatric disorders.

## Abbreviations Used

ACE: adverse childhood experience
ADHD: attention-deficit/hyperactivity disorder
BCPP: board certified psychiatric pharmacist
BPS: Board of Pharmacy Specialties
CMM: comprehensive medication management
MH: mental health
MTP: medication therapy problem
SDOH: social determinants of health
SMI: serious mental illness
SUD: substance use disorder
WHO: World Health Organization
